# Survival Impact of Isocitrate Dehydrogenase (IDH)-Wildtype Histological Versus Molecular Glioblastoma: A Propensity Score-Matched Analysis

**DOI:** 10.7759/cureus.88667

**Published:** 2025-07-24

**Authors:** Nikunj Patil, Sheen Dube, Florence Mutua, Saranya Kakumanu, Jai Jai Shankar, Namita Sinha, Vibhay Pareek

**Affiliations:** 1 Radiation Oncology, CancerCare Manitoba, Winnipeg, CAN; 2 Biochemistry, University of Winnipeg, Winnipeg, CAN; 3 Radiology, University of Manitoba, Winnipeg, CAN; 4 Pathology, University of Manitoba, Winnipeg, CAN

**Keywords:** brain tumor, mgmt, molecular glioblastoma, propensity score match analysis, radiotherapy (rt)

## Abstract

Introduction

Glioblastoma (GBM) is a highly aggressive brain tumor with a poor prognosis. Molecular classification has redefined GBM subtypes, but survival differences between histological GBM (h-GBM) and molecular GBM (mol-GBM) remain underexplored. This study uses propensity score matching (PSM) to compare survival outcomes, accounting for clinical confounders.

Methods

A retrospective cohort of isocitrate dehydrogenase (IDH)-wildtype GBM patients was analyzed using 1:1 nearest-neighbor PSM, balancing age (<64 or ≥64 years), O6-methylguanine-DNA methyltransferase (MGMT) methylation status, and radiotherapy (RT) dose. Kaplan-Meier estimates and log-rank tests were used to compare the overall survival (OS) and progression-free survival (PFS) between h-GBM and mol-GBM.

Results

The matched cohort included 26 h-GBM and 26 mol-GBM patients. Median OS was 15.2 months for mol-GBM vs. 14.2 months for h-GBM (p=0.238). Mol-GBM showed significantly longer PFS (11.8 vs. 8.0 months, p=0.005). In MGMT-unmethylated patients, mol-GBM had superior OS (15.2 vs. 12.7 months, p=0.030) and PFS (13.1 vs. 7.8 months, p<0.001). Higher RT dose (60 Gy/30 fractions) improved OS (22.1 vs. 13.3 months, p=0.010) and PFS (10.8 vs. 8.7 months, p=0.020) in mol-GBM.

Conclusion

Molecular classification significantly influences GBM prognosis, with mol-GBM demonstrating better PFS and selective OS benefits in MGMT-unmethylated patients. Higher RT doses enhance outcomes, supporting personalized treatment strategies. Validation in larger cohorts is needed.

## Introduction

The annual incidence of primary brain tumors worldwide is 308,102, with an annual mortality of 251,329, as per the Global Cancer Observatory (GLOBOCAN) 2020 [1]. Among these primary brain tumors, glioblastoma (GBM) is the most common malignant brain tumor, with a five-year survival of only 10.3% [2], and has been historically treated with surgery followed by concurrent chemoradiation and adjuvant chemotherapy [3]. Recent studies [4-6] have recognized a subset of isocitrate dehydrogenase (IDH)-wildtype diffuse or anaplastic astrocytoma that could be considered as World Health Organization (WHO) grade II or III based on histological features. These include mitosis rate, cellular atypia, absence of microvascular proliferation or necrosis, which have been found to have a poor clinical course, with overall survival similar to IDH-wildtype glioblastoma, WHO grade IV [4-6]. The Consortium to Inform Molecular and Practical Approaches to CNS Tumor Taxonomy - Not Official WHO (cIMPACT-NOW) evaluated the literature to determine and define molecular criteria reliably identifying these tumors that behaved aggressively [6,7]. In their 2019 update, cIMPACT-NOW presented the molecular criteria for diagnosing these aggressive tumors [8]. They concurred that histologic IDH-wildtype diffuse astrocytoma of WHO grade II or III that carry a telomerase reverse transcriptase (TERT) promoter (TERTp) mutation, or an epidermal growth factor receptor (EGFR) amplification, or a whole chromosome 7 gain combined with a whole chromosome 10 loss (+7/-10) are associated with poor patient survival.

Following the publication of this update, survival outcomes were validated in a retrospective cohort study of patients with aggressive IDH-wildtype diffuse astrocytomas [9]. The median survival of IDH-wildtype astrocytomas with TERTp mutation was 14.4 months (p=0.89), similar to that of patients with IDH-wildtype glioblastoma. Various genetic alterations in WHO grade IV glioblastoma have been assessed, including IDH gene mutations and mutations in the promoter region of the TERT gene [10,11]. TERTp mutations have been identified in a subpopulation of GBM and were revealed to be significantly associated with poor clinical prognosis [12,13]. The WHO published an updated CNS 5 classification of central nervous system (CNS) tumors in 2021 [14] and classified IDH-wildtype diffuse astrocytoma with these molecular features as glioblastoma. As the diagnosis was made on the basis of molecular features, they are addressed as molecular GBM (mol-GBM).

Prior studies have indicated that the overall survival of mol-GBM patients is comparable to that of histological GBM (h-GBM) [15-17]. Having said that, the various factors associated with clinical outcomes in mol-GBM are inadequately characterized and have not been thoroughly investigated. We hypothesized that mol-GBM would exhibit different survival outcomes than h-GBM when matched for key clinical and treatment variables. The primary objective was to evaluate the survival difference between mol-GBM and h-GBM. The secondary objective was to assess the impact of MGMT (O6-methylguanine-DNA methyltransferase) status and radiotherapy dose on outcomes. We conducted a propensity score matching (PSM) analysis to evaluate survival differences between both the groups.

## Materials and methods

The clinical records, including the imaging features, of patients with IDH-wildtype diffuse astrocytoma and molecular features of glioblastoma (mol-GBM) and WHO CNS grade 4 h-GBM treated at our institute between January 2015 and June 2023 were retrospectively assessed. Patients aged >18 years, with histopathologically confirmed mol-GBM with IDH-wildtype diffuse astrocytoma but without high-grade histological features such as microvascular proliferation and necrosis, with TERTp mutation or EGFR amplification or concurrent gain of chromosome 7 and loss of chromosome 10 mutation and grade 4 h-GBM were included. The TERTp and EGFR amplification was detected with next-generation sequencing (NGS), whereas concurrent gain of chromosome 7 and loss of chromosome 10 mutation was detected with chromosomal microarray (CMA). Patients were excluded from the study if they had received palliative treatment, did not undergo adjuvant intent chemoradiation therapy, or lacked follow-up imaging or any pertinent follow-up information. The adjuvant treatment included external beam radiotherapy at a dose of 60 Gy in 30 fractions (2 Gy per fraction) or 40 Gy in 15 fractions (2.6 Gy per fraction) with concurrent temozolomide (75 mg/m^2^) following surgical resection or stereotactic biopsy of the tumor. As part of the radiation therapy planning, CT simulation and contouring were done according to the European Society for Radiotherapy and Oncology (ESTRO) guidelines [18]. Patients received adjuvant temozolomide (150-200 mg/m^2^) after completion of radiation. If recurrence was diagnosed on follow-up clinical imaging, further lines of chemotherapy were administered. As part of the follow-up, the patients were assessed with a brain MRI every three months or earlier if they presented clinically with evidence of disease progression.

The baseline, clinical, and treatment characteristics were recorded, and survival outcomes were assessed. Progression-free survival (PFS) was defined as the time from diagnosis to disease progression, as determined by MRI-based imaging. Overall survival (OS) was defined as the time from diagnosis to the date of death from any cause or date of last contact; patients who were alive at last contact were censored. Disease-specific survival (DSS) was defined as the percentage of people who had not succumbed to the primary brain tumor at the last follow-up. MR characteristics such as tumor location, enhancing volume, necrosis, multifocality, and minimum apparent diffusion coefficients were collected. Histopathological parameters such as location, monoclonal antibody immunoblot 1 proliferation (MIB) index, and status of various molecular markers were evaluated. Performance status was not consistently recorded due to the retrospective design and is acknowledged as a limitation.

Statistical analysis

The descriptive analysis of clinical and treatment characteristics was performed using frequency (number and percentage), mean (standard deviation (SD)), and median (range). Statistical analysis was performed using R (R version 4.4.2, The R Foundation, Vienna) and Stata (version 15; StataCorp, College Station, TX). PSM was performed to balance the distribution of selected covariates between patients with h-GBM or mol-GBM and reduce the confounding effects between both groups. The propensity score was estimated using logistic regression analysis with the modeling variables age (categorized as <64 years and ≥64 years), MGMT status (classified unmethylated, or methylated), and radiation dose (60 Gy in 30 fractions and 40 Gy in 15 fractions). One-to-one nearest neighbor matching was employed to achieve a balance between groups. Patients with unknown MGMT methylation status were excluded from stratified and PSM analysis. Kaplan-Meier survival analysis was used to estimate PFS, OS, and the log-rank test was used to compare the curves. A p-value of <0.05 was considered statistically significant.

## Results

Patient characteristics

A total of 32 patients with the diagnosis of m-GBM were identified. Of these, five who received palliative whole-brain irradiation and one patient who received adjuvant chemoradiation at the dose of 54 Gy in 30 fractions were excluded, resulting in 26 patients in the final analysis. Additionally, 165 patients with h-GBM met the eligibility criteria and were included for comparison.

The median age was 62 years (41-78 years) for the mol-GBM group and 64 years (33-85 years) for the h-GBM group. In the mol-GBM group, 25 (96%) patients harbored TERTp mutation, with additional chromosome +7/-10 in one patient (4%). Safe maximal resection was achieved in only five (19%) of mol-GBM cases compared to 138 cases (84%) with h-GBM. Radiological enhancement was seen in 12 (46%) patients compared to 165 (100%) for mol-GBM and h-GBM, respectively. Notably, none of the mol-GBM patients showed evidence of necrosis in contrast to 165 (100%) with h-GBM. Table 1 shows the patient and tumor characteristics.

**Table 1:** The patient, tumor, and treatment characteristics TERTp: Telomerase reverse transcriptase promoter; MGMT: O6-methylguanine-DNA methyltransferase

	Unmatched, n (%)		Matched, n (%)	
Characteristics	Molecular GBM (n=26)	Histological GBM (n=165)	Molecular GBM (n=26)	Histological GBM (n=26)
Median age (range)	62 years (41-78 years)	64 years (33-85 years)	<64: 58 years (41-63 years), ≥64: 72 years (64-78 years)	<64: 59 years (43-62 years), ≥64: 69 years (65-78 years)
Sex				
Male	18 (69%)	105 (64%)	18 (69%)	19 (73%)
Female	8 (31%)	60 (36%)	8 (31%)	7 (27%)
Location				
Temporal	8 (31%)	72 (44%)	8 (31%)	8 (31%)
Frontal	10 (38%)	51 (31%)	10 (38%)	11 (42%)
Occipital	1 (4%)	35 (21%)	1 (4%)	0 (0%)
Parietal	7 (27%)	7 (4%)	7 (27%)	7 (27%)
MGMT status				
Methylated	7 (27%)	51 (31%)	7 (27%)	7 (27%)
Unmethylated	19 (73%)	69 (42%)	19 (73%)	19 (73%)
Unknown	0	45 (27%)	0	0
Molecular markers				
TERTp mutation	25 (96%)	-	25 (96%)	-
Chr 7+/10-	1 (4%)	-	1 (4%)	-
Dose				
60/30	14 (54%)	113 (68%)	14 (54%)	14 (54%)
40/15	12 (46%)	52 (32%)	12 (46%)	12 (46%)
Surgery				
Biopsy	21 (81%)	27 (16%)	21 (81%)	25 (96%)
Resection	5 (19%)	138 (84%)	5 (19%)	1 (4%)
Radiological features				
Enhancement	12 (46%)	165 (100%)	12 (46%)	26 (100%)
Multifocality	5 (19%)	19 (12%)	5 (19%)	5 (19%)
Hemorrhage	2 (8%)	3 (1.2%)	2 (8%)	0 (0%)
Necrosis	0	165 (100%)	0	26 (100%)

Survival analysis between unmatched groups

**Figure 1:**
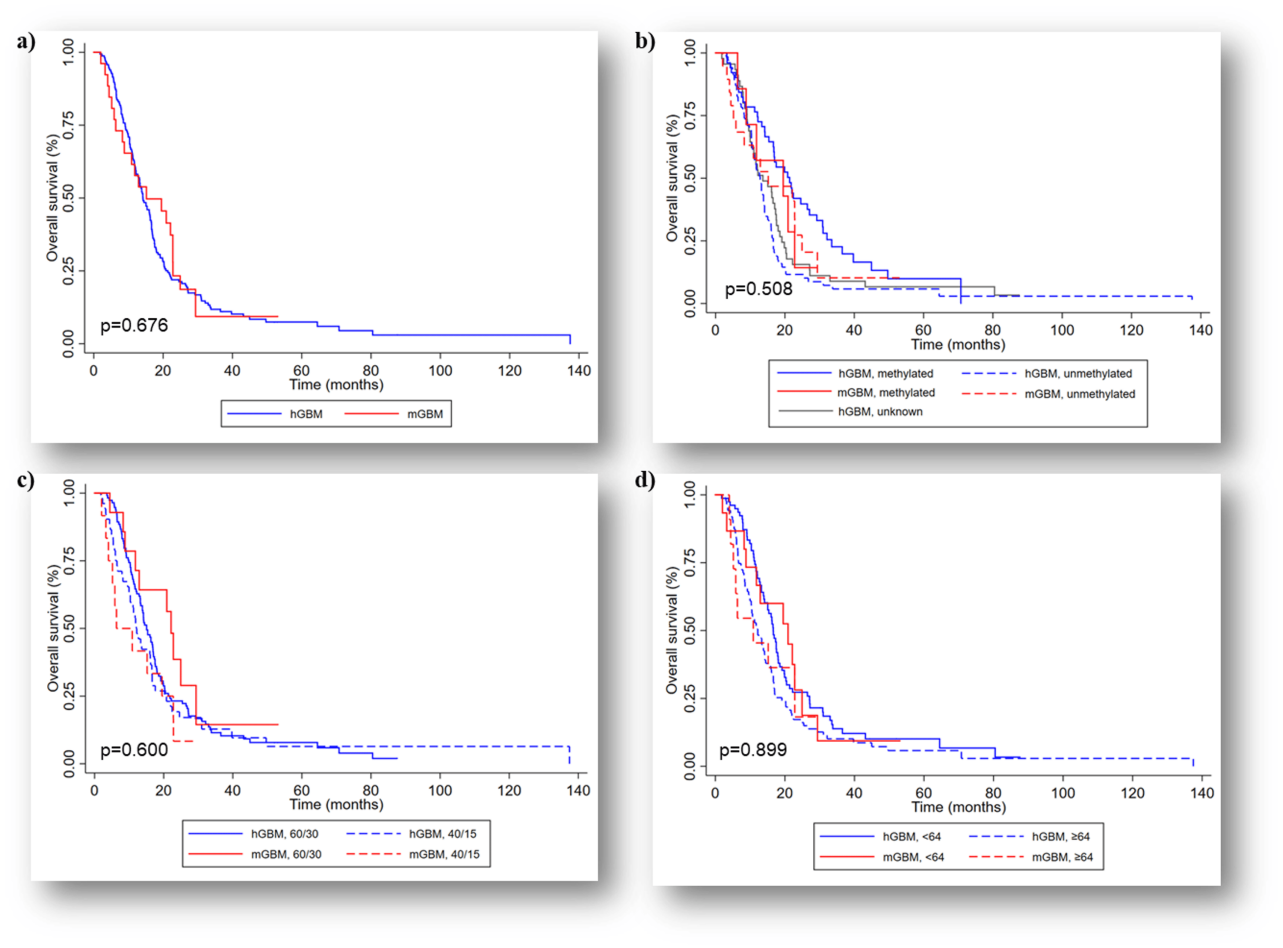
Overall survival (OS): (a) Kaplan-Meier curve showing no difference in median OS in the unmatched study population (p=0.676), (b) Kaplan-Meier curve showing no difference in median OS when stratified when stratified by MGMT status (p=0.508), (c) Kaplan-Meier curve showing no difference in median OS when stratified by the radiotherapy dose (p=0.600), and (d) Kaplan-Meier curve showing no difference in median OS when stratified by median age (64 years) (p=0.899). hGBM: Histological glioblastoma, mGBM: molecular glioblastoma, MGMT: O6-methylguanine-DNA methyltransferase; 60/30: 60 Gy in 30 fractions, 40/15: 40 Gy in 15 fractions.

The median follow-up was 14.2 months (range 1.8-137.5 months). The median OS was better in the mol-GBM group (15.2 months) compared to h-GBM (14.2 months); however, this was not statistically significant (p=0.676) (Figure 1a). The one-year survival rates were 57.7% for mol-GBM and 60.6% for h-GBM, while the two-year survival rates were 24% and 21.3%, respectively. There was no significant difference in OS between the two groups in terms of the MGMT status (p=0.508), age (p=0.899), or dose of radiation (p=0.600) (Figure 1b-d).

**Figure 2:**
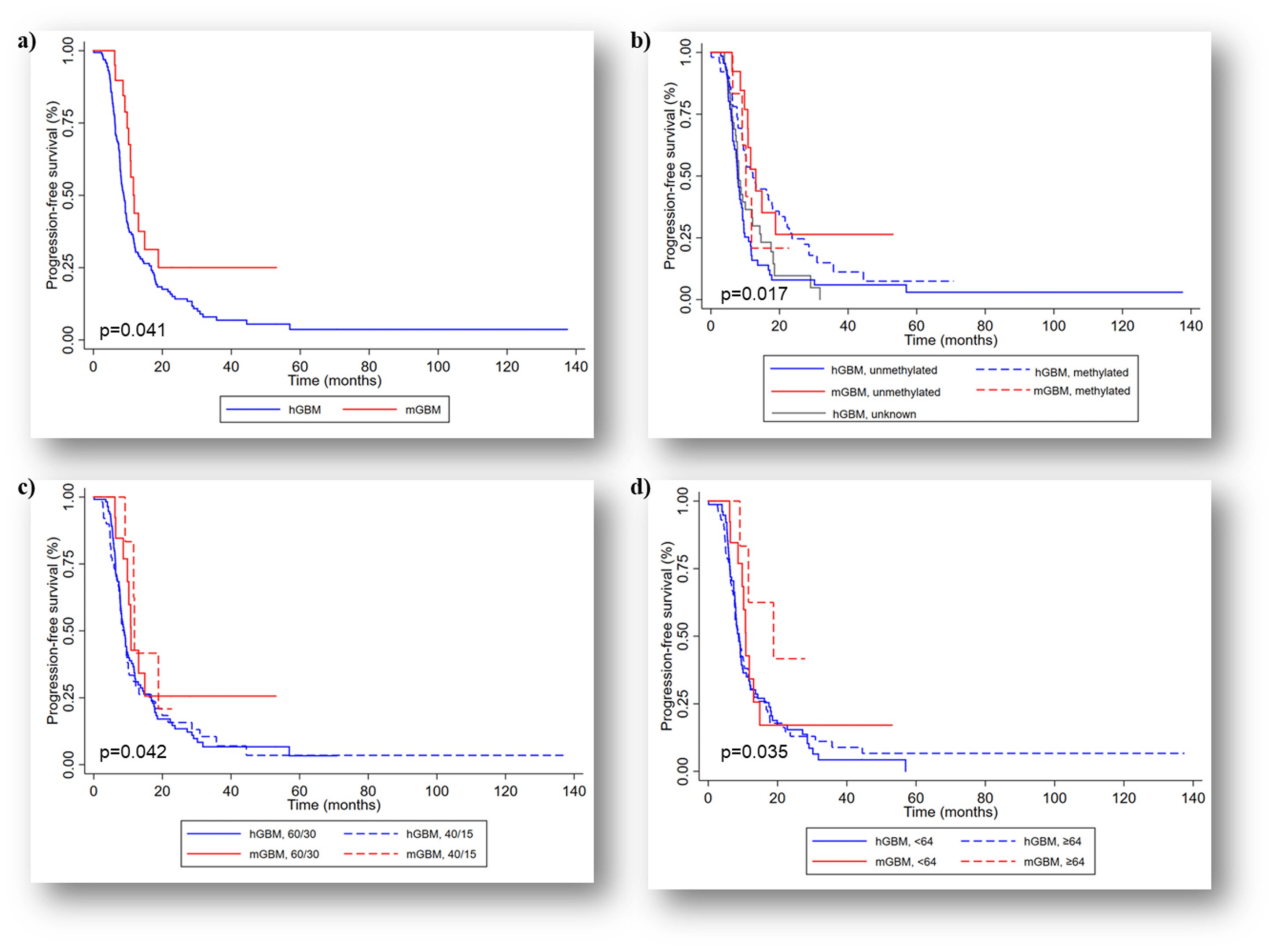
Progression-free survival (PFS): (a) Kaplan-Meier curve showing significant difference in median PFS within the unmatched study population (p=0.041); (b) Kaplan-Meier curve showing significant difference in median PFS stratified by MGMT status (p=0.017), (c) Kaplan-Meier curve showing significant difference in median PFS stratified by the radiotherapy dose, higher dose of radiation was associated with better outcomes (p=0.402), and (d) Kaplan-Meier curve showing significant difference in median PFS stratified by median age (64 years) (p=0.035). hGBM: Histological glioblastoma, mGBM: molecular glioblastoma, MGMT: O6-methylguanine-DNA methyltransferase; 60/30: 60 Gy in 30 fractions, 40/15: 40 Gy in 15 fractions

The mol-GBM group demonstrated a significantly longer median PFS of 11.8 months compared to 8.8 months in the h-GBM group (p=0.041) (Figure 2a). The one-year PFS was 43.2% for mol-GBM and 32.6% for h-GBM, and the two-year PFS were 24.7% and 14.2%, respectively. Mol-GBM group showed a significant difference in PFS compared to h-GBM in terms of the MGMT status (p=0.017), age (p=0.035), or dose of radiation (p=0.042) (Figure 2b-d). Further stratification by MGMT status (unmethylated vs methylated), radiation dose (60 Gy in 30 fractions vs 40 Gy in 15 fractions), and age (<64 years vs ≥64 years) revealed significant difference in PFS of MGMT unmethylated patients with 13.1 months in the mol-GBM group vs 7.8 months in h-GBM (p=0.005), and in patients aged ≥64 years with 18.8 months and 8.9 months, respectively (p=0.04).

Propensity-matched analysis

**Figure 3:**
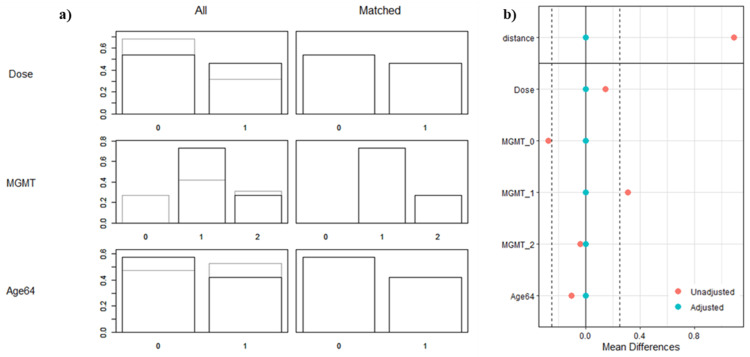
Propensity score distribution balance. (a) Density plots illustrate the distribution of covariates before (all) and after (matched) adjustment. (b) The figure illustrates the balance of the covariates used in the model - MGMT, median age, and dose - before and after propensity score weighting adjustment. Key: Dose: 0=60/30, 1=40/15; MGMT: 0=unknown, 1=unmethylated, 2=methylated; Age 64: 0=<64 years, 1=≥ 64 years. MGMT: O6-methylguanine-DNA methyltransferase, 60/30: 60 Gy in 30 fractions, 40/15: 40 Gy in 15 fractions.

All the 26 mol-GBM patients (100%) were matched to 26 of the h-GBM patients (15.8%) based on the three selected modeling variables - MGMT status, radiation dose and the median age (64 years) of the study population (Figure 3). A satisfactory balance was achieved, with all standardized mean differences remaining below 0.1. The patient, tumor, and treatment characteristics are outlined in Table 1.

**Figure 4:**
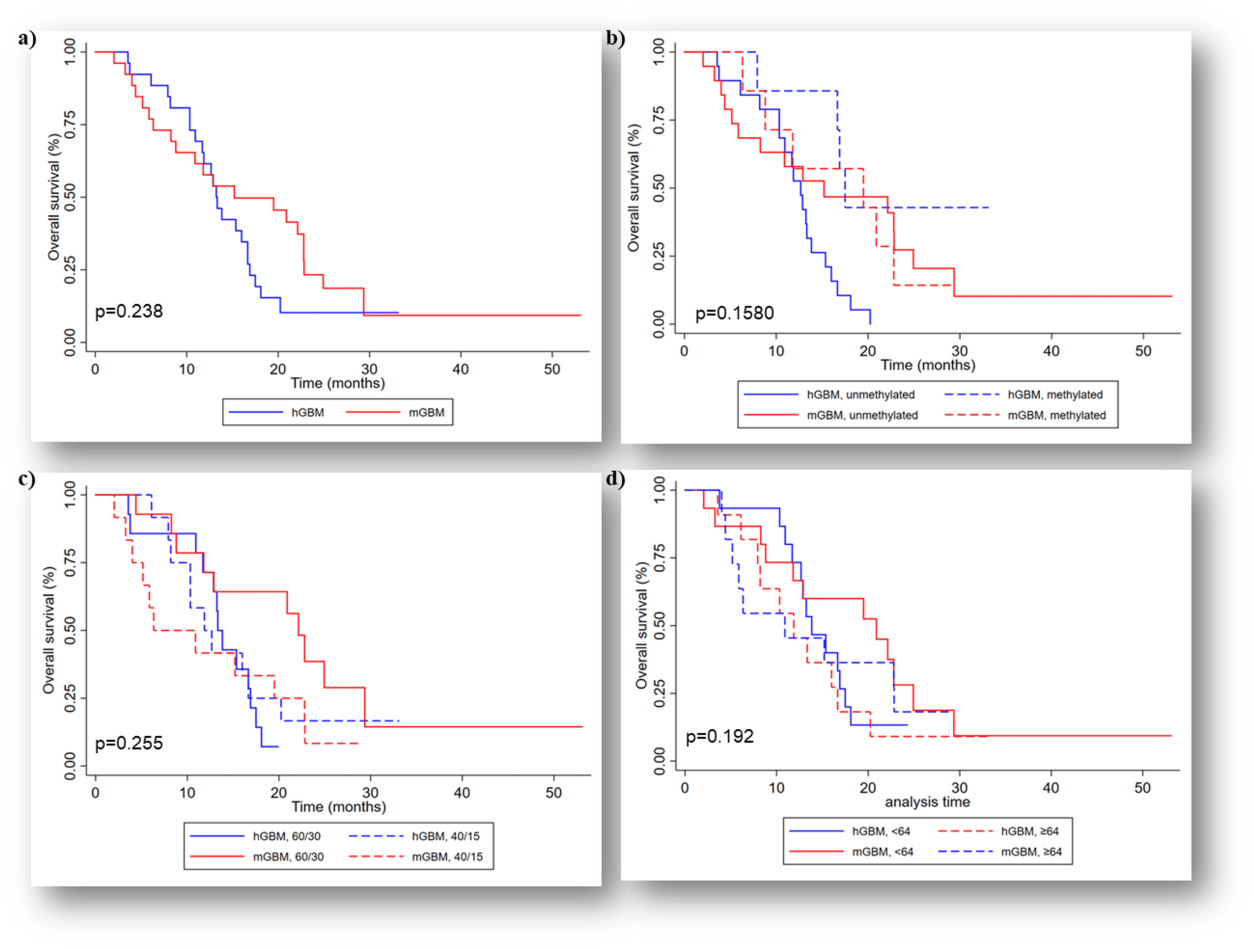
Overall survival (OS): (a) Kaplan-Meier curve showing no difference in median OS in the matched cohort (p=0.238); (b) Kaplan-Meier curve showing no difference in median OS when stratified by MGMT status, the MGMT status showed no difference in OS within mol-GBM cohort (p=0.158); (c) Kaplan-Meier curve showing no difference in median OS when stratified by the radiotherapy dose, patients receiving 60 Gy had better OS (p=0.255), and (d) Kaplan-Meier curve showing no difference in median OS when stratified by median age (64 years), patients <64 years had better OS in both cohorts (p=0.192). hGBM: Histological glioblastoma, mGBM: molecular glioblastoma, MGMT: O6-methylguanine-DNA methyltransferase; 60/30: 60 Gy in 30 fractions, 40/15: 40 Gy in 15 fractions

The median OS was better in the mol-GBM group (15.2 months) compared to h-GBM (14.2 months); however, this was not statistically significant (p=0.238). The one-year survival rates were 57.7% for mol-GBM and 61.5% for h-GBM, while the two-year survival rates were 24% and 10.3%, respectively (Figure 4a). There were no significant differences overall in OS between the groups when stratified by MGMT status (p=0.158) (Figure 4b). Nevertheless, mol-GBM with MGMT unmethylated demonstrated a better OS of 15.2 months compared to 12.7 months in the h-GBM group (p=0.03). Further comparison of the MGMT status (unmethylated vs. methylated) revealed a significant difference in h-GBM (p=0.005) but not in mol-GBM (p=0.860). No significant differences in the OS between mol-GBM and h-GBM using the median age cut-off of 64 years (p=0.192) (Figure 4c). Patients younger than 64 years exhibited better OS than those aged 64 and above in both groups. Furthermore, no significant differences were observed in the OS between the groups when stratified by dose of radiation (p=0.255) (Figure 4d). However, mol-GBM who received 60 Gy in 30 fractions showed a better OS of 22.1 months compared to h-GBM with 13.3 months (p=0.01).

**Figure 5:**
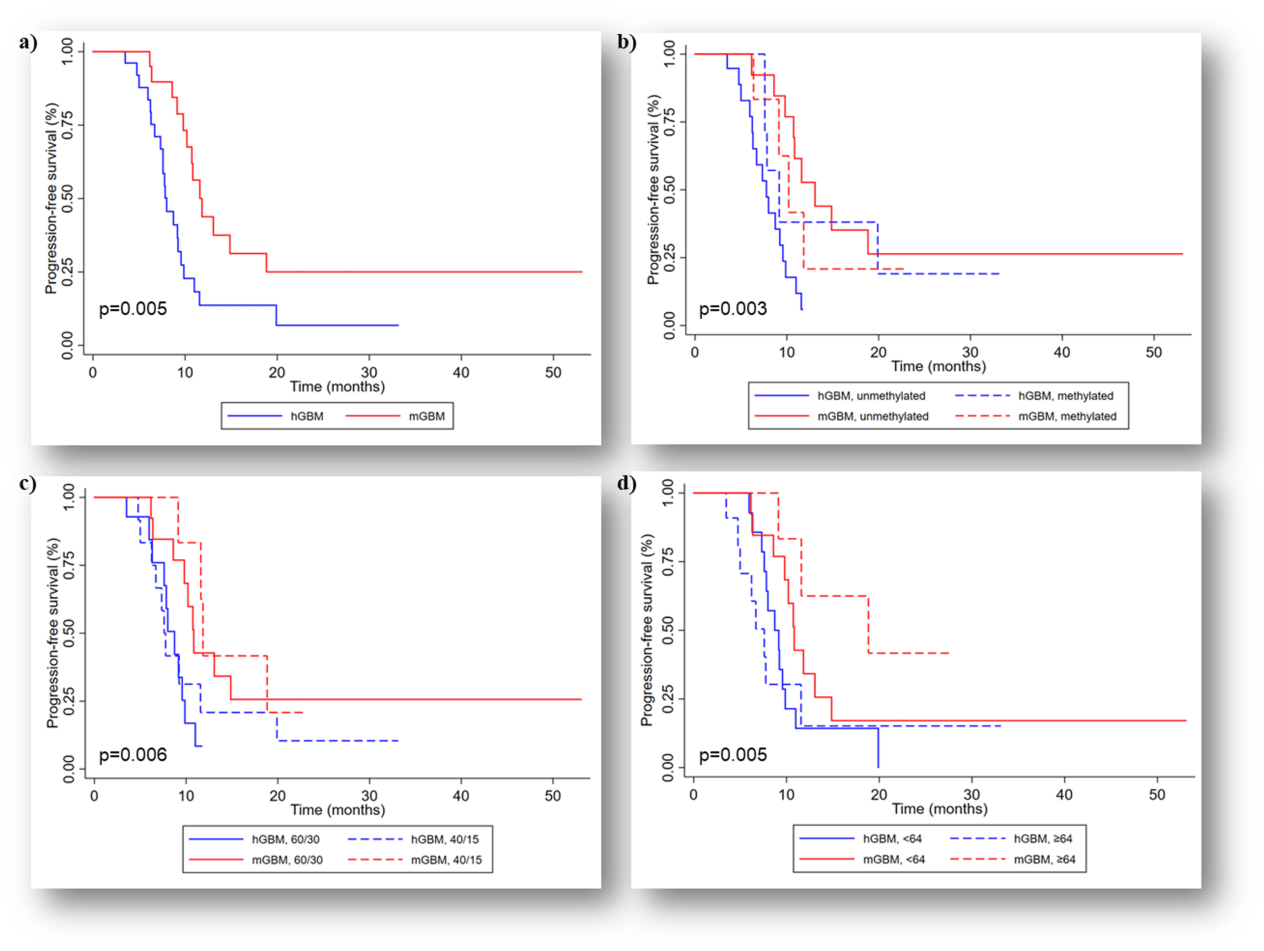
Progression-free survival (PFS): (a) Kaplan-Meier curve showing better median PFS in mol-GBM cohort compared to h-GBM in the matched analysis (p=0.005); (b) Kaplan-Meier curve showing significant difference in median PFS stratified by MGMT status, mol-GBM cohort with MGMT unmethylated tumor had better PFS in comparison to h-GBM cohort (p=0.003); (c) Kaplan-Meier curve showing significant difference in median PFS stratified by the radiotherapy dose, higher dose of radiation was associated with better outcomes (p=0.006), and (d) Kaplan-Meier curve showing significant difference in median PFS stratified by median age (64 years), mol-GBM patients ≥64 years of age had better PFS than the matched h-GBM cohort (p=0.005). hGBM: Histological Glioblastoma, mGBM: Molecular Glioblastoma, MGMT: O6-methylguanine-DNA methyltransferase, 60/30: 60 Gy in 30 fractions, 40/15: 40 Gy in 15 fractions

The mol-GBM group demonstrated a significantly longer median PFS of 11.8 months compared to 8.0 months in the h-GBM group (p=0.005) (Figure 5a). The one-year PFS was 43.2% for mol-GBM and 12.5% for h-GBM, and the two-year PFS were 24.7% and 6.2%, respectively. There was a significant difference overall in PFS between the groups when stratified by MGMT status (p=0.003) (Figure 5b). The mol-GBM patients with MGMT unmethylated demonstrated a better PFS of 13.1 months compared to 7.8 months in the h-GBM group (p<0.001). Further comparison of the MGMT status (unmethylated vs. methylated) revealed no difference in the PFS of h-GBM (p=0.15) or mol-GBM (p=0.51). There was a significant difference overall in PFS between mol-GBM and h-GBM using the median age cut-off of 64 years (p=0.005) (Figure 5c). The PFS of patients <64 years showed no significant difference with mol-GBM (10.8 months) versus h-GBM (8.7 months) (p=0.07) but differed significantly in the ≥64 years group with PFS of 18.8 months and 7.6 months, respectively (p=0.02). Lastly, a significant difference overall in PFS between the groups when stratified by radiation dose (p=0.006) (Figure 5d). mol-GBM who received 60 Gy in 30 fractions showed a better PFS of 10.8 months than h-GBM with 8.7 months (p=0.02).

Treatment on the progression of the disease

Magnetic resonance (MR) spectroscopy and perfusion imaging were used to confirm the progression of the disease. All the cases were discussed in a multidisciplinary tumor board before proceeding with treatment. A total of 11 (42%) mol-GBM and 85 (52%) h-GBM patients received second-line treatment, with lomustine the most common second-line drug used in both groups. Three (11%) mol-GBM and 35 (21%) h-GBM patients received third-line therapy after progression was observed while on the second-line treatment. Overall, seven patients (3.6%) received reirradiation either with external beam therapy or Gamma knife radiosurgery. Treatment on the progression of the disease is outlined in Table 2.

**Table 2:** Treatment received on progression of disease GBM: Glioblastoma.

Treatment on progression:	Molecular GBM, n (%)	Histological GBM, n (%)
Second line:		
Lomustine	8 (72.7%)	43 (50.5%)
Bevacizumab	2 (18.2%)	12 (14%)
Carboplatin and Tamoxifen	1 (9.1%)	0
Surgery and Lomustine	0	8 (9.4%)
Surgery and Temozolomide	0	6 (7%)
Surgery and Carboplatin with Tamoxifen	0	1 (1.4%)
Surgery and Bevacizumab	0	2 (2.3%)
Reirradiation and temozolomide	0	2 (2.3%)
Gamma knife radiosurgery	0	2 (2.3%)
Temozolomide	0	8 (9.4%)
Reirradiation	0	1 (1.4%)
Third line:		
Bevacizumab	1 (33.3%)	11(31.4%)
Lomustine	0	11 (31.4%)
Carboplatin and Tamoxifen	1 (33.3%)	2 (5.8%)
Surgery	1 (33.4%)	3 (8.5%)
Etoposide	0	6 (17.1%)
Reirradiation	0	2 (5.8%)
Fourth line:		
Bevacizumab	0	4 (33.4%)
Lomustine	0	1 (8.3%)
Etoposide	0	1 (8.3%)
Carboplatin and Tamoxifen	0	6 (50%)
Fifth line:		
Bevacizumab	0	4 (100%)

## Discussion

The WHO made significant revisions in the classification of CNS tumors published in the fifth edition in 2021 [14]. These changes categorizing CNS tumors show the emergence of mol-GBM as a new entity. These IDH-wildtype diffuse astrocytoma were reclassified as WHO CNS grade 4 glioblastoma to reflect their aggressive clinical behavior, which closely parallels that of histologically defined glioblastoma. However, mol-GBM presents distinct clinical and radiological features from h-GBM. The prognostic factors that impact the survival of mol-GBM are understudied. Here, we present data on the clinical outcomes of mol-GBM and h-GBM, as well as the impact of various prognostic factors on the survival of patients who received curative treatment. We observed significant differences between the groups in PFS, but not in OS, with better PFS in mol-GBM in both the analysis of the unmatched and 1:1 propensity score-matched cohorts of the study population. Notably, mol-GBM also showed higher OS and PFS in patients with unmethylated MGMT status and patients on higher RT doses.

The landmark study by Stupp et al. [3] demonstrated a median OS of 14.6 months in patients with h-GBM receiving adjuvant chemoradiation versus radiation [3]. In our matched analysis, the median OS was 15.2 months for mol-GBM and 14.2 months for the h-GBM group. Although the difference was not statistically significant, mol-GBM exhibited slightly better survival despite a higher incidence of multifocal disease and undergoing less extensive surgery. Notably, the OS improved to 22.1 months in mol-GBM if they received a higher radiation dose, compared to 13.3 months in h-GBM. A recently published propensity-matched analysis [19] showed that mol-GBM (36.1 months) had a superior survival compared to h-GBM (15.7 months) (p<0.001). Their outcomes were notably higher than those observed in our study, which could be potentially explained by more patients receiving extensive surgery (39, 75%) vs (5, 19%) in our cohort, as well as a longer median follow-up in their study.

Several prognostic factors are known to impact the outcomes of h-GBM patients, including MGMT methylation status, age, extent of surgery, performance status, and tumor location [20-22]. It is well established that MGMT promoter methylation status is one of the most prognostic biomarkers. The epigenetic silencing of MGMT by promoter methylation leads to reduced DNA repair and increased sensitivity to temozolomide [23-25]. The European Organization for Research and Treatment of Cancer - National Cancer Institute of Canada Clinical Trials Group (EORTC-NCIC) trial [25] evaluated the prognostic implications of MGMT promoter methylation in patients with h-GBM and found that patients who received adjuvant chemoradiation had a two-year survival of 48.9% and 14.8% for the MGMT methylated and unmethylated groups, respectively. In our matched analysis, when we compared the OS based on MGMT status (unmethylated vs methylated), there was a significant difference within the h-GBM (p=0.005) but not within the mol-GBM group (p=0.860). Further analysis showed that the median OS and PFS were superior in the MGMT unmethylated mol-GBM group compared to the unmethylated h-GBM. A recent retrospective study [26] concluded no difference in OS between mol-GBM and h-GBM (p=0.435), but mol-GBM was associated with better PFS (p=0.023). On multivariable analysis, MGMT did not impact outcomes in the mol-GBM cohort. These results reflect our study findings as well. All the above findings suggest that the tumors behave differently, suggesting a separate approach in management.

Several studies have reported a strong association between young age and long-term clinical outcomes [27-29]. This is in agreement with our study, in which younger patients (<64 years) showed a more favorable OS within both cohorts. A systematic review and meta-analysis [30] evaluated the outcomes of patients with mol-GBM and prognostic factors that impacted survival [30] found that patients with mol-GBM with grade II disease did better than h-GBM (p=0.014), whereas patients with grade III disease showed survival patterns identical to h-GBM. They identified that young age, extent of surgery, and grade (II vs III) were associated with better outcomes. Moreover, they suggested that the MGMT status did not impact survival. These findings are similar to our findings; we did not capture data on tumor grade in our review and did not study the impact of the extent of surgery on clinical outcomes.

A recent study by Wijnenga et al. [31] evaluated the outcomes in patients with mol-GBM and found that the median OS in patients with grade II glioma was 17 months versus 18 months in grade III or IV. Isolated TERTp mutation had a median OS of 17 months versus 21 months in patients with EGFR amplification and/or Chr 7+/10-. These findings conflicted with the results found by Berzero et al. [32], suggesting additional studies would be necessary to study the impact of pathological grade and various molecular features on outcomes. We did not study these factors in our study.

The Canadian phase II trial [33] and then the EORTC CCTG CE.6 trial [34] established that hypofractionated adjuvant chemoradiation had equivalent clinical outcomes in comparison to conventional chemoradiation in old and frail patients. The prognostic implication of radiation dose has not been studied in the mol-GBM in the existing literature. Our study provides insight and highlights that a higher radiation dose is associated with better OS and PFS within the mol-GBM cohort.

Radiologically, mol-GBM patients present with non-enhancing, infiltrative lesions resembling low-grade gliomas [9,30,35-38] in contrast to contrast-enhancing, necrotic, well-defined appearance more commonly seen with h-GBM [30,39]. These patients usually present with gyriform involvement involving eloquent structures like the thalamus, and hence, most patients undergo biopsy rather than resection [9,37]. This is consistent with our findings, with 21 (81%) patients undergoing biopsy as opposed to 27 (16%) with h-GBM. Our mol-GBM cohort showed that 12 (46%) patients showed contrast enhancement, 5 (19%) had multifocal disease, and none of them showed any evidence of necrosis on imaging. Patients with mol-GBM have a higher incidence of seizures [40] compared to h-GBM and a longer median time from onset of seizure to diagnosis [40]. It is a well-known fact that low-grade gliomas usually present with symptoms of seizures [41], and mol-GBM is considered histologically low-grade. Contrary to this histological classification, the presence of specific molecular markers classifies them as high-grade.

The main strength of this study was that we were able to assess the outcomes in the entire study population and compare these between mol-GBM and h-GBM in a propensity score-matched subset of the study population. Our findings are consistent with the literature, conforming to it in that the overall prognosis of patients with mol-GBM remains better than that of patients with h-GBM. Moreover, our study helps to bridge the knowledge gap by highlighting the prognostic implications of radiation dose and MGMT status amongst this group. Prospective, multi-institutional studies with larger cohorts are needed to validate these findings. Additionally, future work could incorporate radiomics and genomic profiling, which could elucidate differences between the two cohorts, further refine diagnostic accuracy and support precision treatment approaches. 

The study limitations included the relatively small number of patients from a single centre, short follow-up duration, and a retrospective study design. Owing to the retrospective nature of the study, comprehensive clinical data were not captured, limiting our ability to evaluate the impact of the extent of surgery, performance status, grade, and presence of seizures on clinical outcomes. We did not incorporate emerging quantitative imaging biomarkers, including radiomics or volumetric analysis in our study. There is also a risk of some selection bias that patients with good performance status received 60 Gy as opposed to 40 Gy of radiation. Further, although the use of multiple statistical comparisons may increase type I error risk, there is an added benefit of using PSM, which minimizes confounding.

## Conclusions

This study highlights the prognostic significance of molecular classification in IDH-wildtype GBM. Mol-GBM exhibits longer PFS and selective OS benefits in MGMT-unmethylated patients compared to h-GBM. Higher radiation doses were associated with improved outcomes; however, given the retrospective design and non-randomized allocation, these observations should be interpreted with caution. These findings advocate for a precision medicine approach, tailoring the treatment based on molecular and clinical factors. Larger, multi-institutional studies are essential to validate these results and optimize therapeutic strategies for mol-GBM.
